# Association of CMV-Specific T Cell-Mediated Immunity with CMV DNAemia and Development of CMV Disease in HIV-1–Infected Individuals

**DOI:** 10.1371/journal.pone.0137096

**Published:** 2015-08-31

**Authors:** Maximilian C. Aichelburg, Lukas Weseslindtner, Mattias Mandorfer, Robert Strassl, Armin Rieger, Thomas Reiberger, Elisabeth Puchhammer-Stöckl, Katharina Grabmeier-Pfistershammer

**Affiliations:** 1 Department of Dermatology, Division of Immunology, Allergy and Infectious Diseases (DIAID), Medical University of Vienna, Vienna, Austria; 2 Department of Virology, Medical University of Vienna, Vienna, Austria; 3 Department of Internal Medicine III, Division of Gastroenterology & Hepatology, HIV & Liver Study Group, Medical University of Vienna, Vienna, Austria; 4 Department of Laboratory Medicine, Division of Clinical Virology, Medical University of Vienna, Vienna, Austria; University of Regensburg, GERMANY

## Abstract

**Background:**

Among HIV-1–infected individuals, cytomegalovirus (CMV) reactivation and disease occur in the setting of advanced immunosuppression. The value of a standardized assessment of CMV-specific T-cell mediated immunity by the CMV QuantiFERON assay (CMV-QFT) has not yet been thoroughly investigated in HIV-1–infected subjects.

**Methods:**

Prospective, longitudinal study in 153 HIV-1–infected subjects with a CD4^+^ T cell count < 350/μL who simultaneously underwent CMV-QFT, CMV serology testing and CMV-DNA quantification. Factors associated with CMV-QFT were evaluated. Clinical screening for CMV manifestations was then performed every 3 months.

**Results:**

Among the 141 CMV IgG-seropositive individuals the CMV-QFT assay yielded reactive results in 84% (118/141), negative results in 15% (21/141) and indeterminate (negative mitogen IFN-gamma response) results in 1% (2/141) of subjects. The mean actual CD4^+^ T cell count was significantly higher in CMV-QFT reactive subjects, when compared to CMV-QFT non-reactive individuals (183 ± 102 vs. 126 ± 104 cells/μL, *P* = 0.015). A significantly lower proportion of CMV-QFT reactive vs. non-reactive patients displayed CMV DNAemia > 100 copies/mL (23% (27/118) vs. 48% (11/23), *P* = 0.02). Furthermore, a statistically significant inverse association between mitogen IFN-gamma response and CMV-DNAemia > 1000 copies/mL was observed (*P* < 0.001). During the observational period, 5 CMV end-organ manifestations were observed. In three of the CMV cases the CMV-QFT yielded indeterminate results.

**Conclusions:**

While CMV-QFT reactivity indicates CMV-specific immunity, indeterminate results due to negative mitogen IFN-gamma response might reflect HIV-1-induced immunodeficiency. Thus, dependency upon CD4^+^ T cell count should be considered when interpreting CMV-QFT results.

## Introduction

Before the introduction of antiretroviral therapy (ART), cytomegalovirus (CMV) infection was one of the clinically most relevant opportunistic infections in individuals with human immunodeficiency virus type 1 (HIV-1) infection [1]. Until then, approximately 40% of HIV-1–infected patients with advanced immunosupression suffered from manifestations of CMV during life-time [2]. The implementation of ART has significantly reduced the risk of CMV reactivation and CMV-related end organ manifestations [3,4].

Retinitis is still the most common manifestation of CMV disease, accounting for about 85% of all cases [5]. CMV retinitis is primarily observed in ART-naive patients who are newly diagnosed with advanced HIV-1 infection and suffer from severe immune impairment (late presenters) [6]. However, manifestations of CMV have also been described in the setting of higher CD4^+^ T cell counts and have been associated with lack of CMV-specific immunity [7]. Individuals with poor CMV-specific immunity may benefit from closer virological monitoring and a lower threshold for pre-emptive antiviral treatment.

Regular virological surveillance coupled with pre-emptive antiviral therapy upon subclinical reactivation is effective in preventing clinical disease and is widely used in individuals on immunosuppression after solid organ transplantation [8]. Assays that detect the production of interferon-gamma (IFN-γ) following stimulation with whole CMV antigens or CMV peptides have previously been used to identify the presence of CMV-specific immunity and have been correlated with protection against CMV reactivation or disease in HIV-1–infected individuals [9]. The CMV QuantiFERON assay (CMV-QFT) is based on ELISA. Similar to the widely used diagnostic test for Mycobacterium tuberculosis [10], the level of IFN-γ, which is mainly produced by specific CD8^+^ T cells, is quantified. In the immunosuppression/transplantation setting the CMV-QFT has been shown to be a useful predictor of spontaneous clearance of low-level viraemia [11]. However, its potential application in HIV-1 infection has as yet not thoroughly been investigated.

The objectives of this prospective, longitudinal study in a cohort of HIV-1–infected individuals with advanced immunosuppression were (i) to assess the association between epidemiological, HIV-1-related and CMV-related factors and CMV-QFT result and (ii) to determine the predictive value of the CMV-QFT for the development of CMV end-organ manifestation.

## Material and Methods

### Study setting and recruitment

This prospective longitudinal study was performed at the Medical University of Vienna, a tertiary center with a HIV clinic. HIV-1-infected individuals aged ≥ 18 years with an actual CD4^+^ T cell count < 350/μl were eligible. Subjects with active CMV disease at baseline were excluded. To provide a real-life assessment of patients, no other exclusion criteria were defined. Consecutive HIV-1-infected patients were enrolled after obtaining written informed consent. All patients were followed longitudinally to assess the development of CMV manifestations for at least 12 months.

### Ethics

The study was approved by the ethics committee of the Medical University of Vienna (EK number 1310/2013). Written informed consent was obtained from all patients prior to the study inclusion.

### Data collection and clinical assessment

Sex, age, ethnicity, country of birth, clinical information on HIV-1–disease including mode of infection, stage of disease according to the CDC [12], CD4^+^ T cell nadir count and antiretroviral therapy (ART) of each subject were collected using the Austrian HIV Cohort Study (AHIVKOS) data management program. Smoking habits and the history of previous CMV disease were assessed using a standardized questionnaire with the assistance of a study nurse if necessary. Smokers were defined as current smokers at study inclusion. All individuals underwent a physical examination for CMV manifestation and referred to the ophthalmologic outpatient clinic for fundoscopy.

### Laboratory assays

Blood samples for CMV DNA load, CMV serostatus and CMV-QFT were drawn after written informed consent. Quantitative assessment of the CMV DNA load in plasma samples was performed by PCR using the Abbott CMV RealTi*me* assay (limit of detection: 20 copies/mL) on an Abbott m2000 platform (Abbott Molecular, Des Plaines, IL, USA). CMV serostatus was assessed using the Diasorin Liaison CMV IgGII chemiluminescent immunoassay (Diasorin, Saluggia, Italy) and primary CMV infection was detected by the Diasorin Liaison CMV IgMII chemiluminescent immunoassay (Diasorin, Saluggia, Italy). CMV-specific T cell-mediated immunity was determined using the Quantiferon-CMV assay (Cellestis Ltd, a QIAGEN company) [13]. A heparinized whole-blood sample was obtained from each study participant, from which aliquots of 1 mL were placed into three seperate tubes. The tubes were then shaken vigorously and incubated overnight at 37°C. The first tube contained a mix of 21 CMV CD8^+^ T cell synthetic peptides derived predominantly from CMV pp65 and IE1 but also included epitopes from pp50, IE2 and gB. The second tube contained the mitogen phytohemagglutinin and served as positive control and the third tube was the negative control and contained sterile phosphate-buffered saline. All patients displayed HLA haplotypes including the epitopes covered by the CMV-QFT assay. After incubation for 24 hours at 37°C, serum supernatants were harvested and kept frozen at -20°C. Batch testing on cryopreserved samples was performed at a median interval of 17.2 days. The tubes were centrifuged and supernatants were recovered. IFN-γ-levels (IU/mL) were measured using an enzyme-linked immunosorbent assay (ELISA) according to the manufacturer’s instructions. A response in the CMV-QFT assay was considered reactive if the IFN-γ response to CMV peptides exceeded the negative control value at least by 0.2 IU/mL. If the mitogen response was lower than 0.5 IU/mL, the test result was interpreted as indeterminate. In case of an indeterminate CMV-QFT result the assay was repeated within one month. The lymphocyte subtypes (CD4^+^ and CD8^+^ T cell counts) and HIV-1 RNA viral load were measured at the time of study inclusion and every three months thereafter.

### Statistical analyses

Statistical analyses were performed using IBM SPSS Statistics 21 (SPSS Inc., Chicago, Illinois, USA). Continuous variables were reported as mean ± standard deviation or median (interquartile range (IQR)), while categorical variables were reported as number of patients with (proportion of patients with) the certain characteristic. Student’s t-test was used for group comparisons of continuous variables when applicable. Otherwise, Mann-Whitney-U test or Kruskal–Wallis one-way analysis of variance were applied. Adjustments for multiple comparisons were performed. Pearson's chi-squared or Fisher’s Exact test were applied for group comparisons of categorical variables. The association between CMV-specific and mitogen IFN-gamma response and both CD4^+^ T cell count and CMV-DNAemia was illustrated using Tukey’s schematic boxplot. All p values (*P*) were two-tailed and *P* < 0.05 was considered to be statistically significant.

## Results

### Baseline characteristics of the study participants

**Fig 1:**
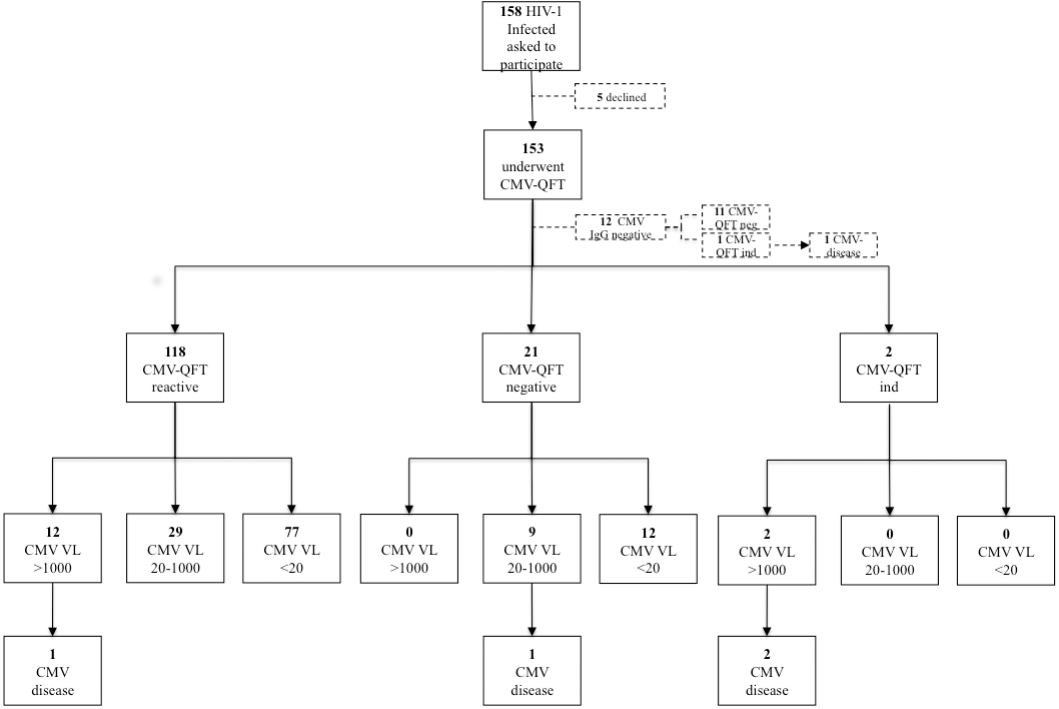
Study profile for HIV-1 infected individuals who underwent CMV-QFT testing. CMV-QFT = Cytomegalovirus-QuantiFERON assay.

One-hundred fifty-eight HIV-1–infected individuals were screened for the inclusion in this study. A total of 153 subjects underwent CMV-QFT testing, as five patients declined participation (Fig 1). The mean age was 43.6 ± 10.6 years, 72% (110/153) of the subjects were male and 28% (43/153) were female. The majority of individuals were born in Europe (82%; 125/153). Eighty subjects (52%) were smokers. Seventy-seven percent (117/153) of patients were currently on ART. HIV-1 RNA levels were below the limit of quantification (< 20 copies/mL) in 34% (52/153) of the subjects, while 24% (36/153) displayed low-level viremia (20–400 copies/mL). The mean actual and nadir CD4^+^ T cell counts were 167 ± 105 and 55 ± 110 cells/μL, respectively. Thirty-nine percent (60/153) of the subjects had previously been diagnosed with an AIDS-defining disease according to the Centers for Disease Control and Prevention classification [12] (Table 1).

**Table 1:** Baseline charactersitics of the study population.

	All subjects	
No. of subjects	153	
Demographics		
Mean age (years)	43.6 ± 10.6	
Sex		
Male	110	(72%)
Female	43	(28%)
Country of origin		
Western Europe	101	(66%)
Eastern Europe	24	(16%)
Africa	19	(12%)
Other	9	(6%)
Smoking	80	(52%)
HIV-1-related factors		
Mode of infection with HIV-1		
Heterosexual contact	56	(37%)
MSM	49	(32%)
IVDU	44	(29%)
Transfusion or unknown	4	(2%)
Mean CD4^+^ T cell count (cells/μL)	167 ± 105	
Mean CD8^+^ T cell count (cells/μL)	625 ± 562	
Mean CD4^+^/CD8^+^ ratio	0.22 ± 0.258	
Mean CD4^+^ T cell count nadir (cells/μL)	55 ± 110	
HIV-1 RNA by strata		
< 20 copies/mL	52	(34%)
20–400 copies/mL	36	(24%)
> 100 000 copies/mL	31	(20%)
On ART, at enrollment	117	(77%)
Previous AIDS-defining manifestation	60	(39%)
CMV-related parameters		
CMV serology		
IgG seropositive	141	(92%)
IgM seropositive	3	(2%)
CMV DNA by strata		
CMV DNA 20–1000 copies/ml	38	(25%)
CMV DNA > 1000 copies/ml	15	(10%)
Previous CMV disease	1	(1%)

AIDS = acquired immunodeficiency syndrome; ART = antiretroviral therapy; CDC = Center for Diseases Control and Prevention; CMV = cytomegalovirus; HIV-1 = human immunodeficiency virus type 1; IVDU = intravenous drug use; MSM = men who have sex with men

The majority of individuals were seropositive for CMV IgG (92%; 141/153). Fifty-three subjects (35%) had CMV-DNAemia (> 20 copies/mL). CMV-related disease, i.e. CMV retinitis, had previously been diagnosed in one patient (Table 1).

### Correlation between CMV QuantiFERON assay and CMV-DNAemia in CMV IgG-seropositive individuals

After exclusion of the 12 subjects with negative CMV IgG serology, the CMV-QFT assay yielded reactive results in 84% (118/141), negative results in 15% (21/141) and indeterminate results in 1% (2/141) of subjects, respectively (Fig 1). All indeterminate results were due to the absence of an adequate mitogen IFN-gamma response. Among the 118 subjects with reactive CMV-QFT, 29 individuals had 20–1000 copies/mL and 12 patients had CMV-DNAemia > 1000 copies/mL, respectively. Nine CMV-QFT negative patients displayed low-level CMV-DNAemia (20–1000 copies/mL). The two individuals with CMV-QFT indeterminate results both had CMV DNA levels > 1000 copies/mL (Fig 1). Forty-eight percent (11/23) of CMV-QFT non-reactive when compared to 23% (27/118) of CMV-QFT reactive subjects had a CMV DNA level > 100 copies/mL (*P* < 0.02) (Table 2).

**Table 2:** Association of risk factors with CMV-QFT reactivity vs. non-reactivity among CMV-IgG-seropositive individuals.

			CMV-QFT results				
	All subjects		Reactive		Non-reactive or indeterminate		*P* value
No. of subjects	141		118		23		
Demographics							
Mean age (years)	43.4 ±10.5		43.9 ±10.7		40.8 ±9.1		0.19
Male sex	103	(73%)	89	(75%)	14	(61%)	0.15
Country of birth							
Western Europe	89	(63%)	77	(65%)	12	(52%)	
Eastern Europe	24	(17%)	22	(19%)	2	(9%)	
Africa	19	(13%)	12	(10%)	7	(30%)	**0.047**
Other	9	(6%)	7	(6%)	2	(9%)	
Smoking	77	(55%)	63	(53%)	14	(61%)	0.51
HIV-1-related factors							
Mode of infection with HIV-1							
Heterosexual contact	50	(35%)	39	(33%)	11	(48%)	0.626
MSM	49	(35%)	43	(36%)	6	(26%)	
IVDU	38	(27%)	32	(27%)	6	(26%)	
Transfusion/unknown	4	(3%)	4	(4%)	0	(0%)	
Mean CD4^+^ T cell count (cells/μL)	173 ± 104		183 ± 102		126 ± 104		**0.015**
CD4^+^ T cell count by strata							
< 50 cells/μL	23	(16%)	15	(13%)	8	(35%)	
50–200 cells/μL	63	(45%)	53	(45%)	10	(43%)	
> 200 cells/μL	55	(39%)	50	(42%)	5	(22%)	
Mean CD8^+^ T cell count (cells/μL)	641 ± 600		672 ± 589		564 ± 588		0.161
Mean CD4^+^/CD8^+^ ratio	0.223 ± 0.264		0.224 ± 0.239		0.116 ± 0.259		0.087
Mean CD4^+^ T cell count nadir (cells/μL)	58 ± 114		60 ± 112		56 ± 106		0.674
Mean HIV-1 RNA (log_10_ copies/ml)	1.86 ± 4.54		1.74 ± 3.77		2.85 ± 5.32		0.147
HIV-1 RNA < 20 copies/mL	49	(30%)	42	(36%)	7	(30%)	0.811
Previous AIDS defining manifestation	53	(38%)	42	(36%)	11	(48%)	0.811
CMV-related parameters							
IgM seropositive	3	(2%)	3	(3%)	0	(0%)	0.584
CMV DNA	0 (132)		0 (89)		0 (567)		0.158
CMV DNA > 100 copies/mL	38	(27%)	27	(23%)	11	(48%)	**0.02**
CMV DNA > 1000 copies/mL	14	(9%)	12	(10%)	2	(9%)	1

ART = antiretroviral therapy; CDC = Center for Diseases Control and Prevention; CMV = cytomegalovirus; HIV-1 = human immunodeficiency virus type 1; IVDU = intravenous drug use; MSM = men who have sex with men

### CMV-QFT results by CD4^+^ T cell counts in CMV IgG-seropositive subjects

The mean actual CD4^+^ T cell count was significantly higher in CMV-QFT reactive subjects when compared to CMV-QFT non-reactive individuals (183 ± 102 vs. 126 ± 104 cells/μL, *P* = 0.015). Among CMV-QFT reactive individuals, 13% (15/118) and 45% (53/118) were in the CD4^+^ T cell count strata of < 50 or 50–200 cells/μL, respectively. In comparison, 35% (8/23) and 43% (10/23) of CMV-QFT non-reactive subjects had CD4^+^ T cell counts of < 50 or 50–200 cells/mm^3^. The CD4^+^ T cell counts were > 200 cells/μL in 42% (50/118) vs. 22% (5/23) of subjects with a reactive and non-reactive CMV-QFT (Table 2).

### Impact of CD4^+^ T cell count on CMV-specific and mitogen IFN-gamma response

**Fig 2:**
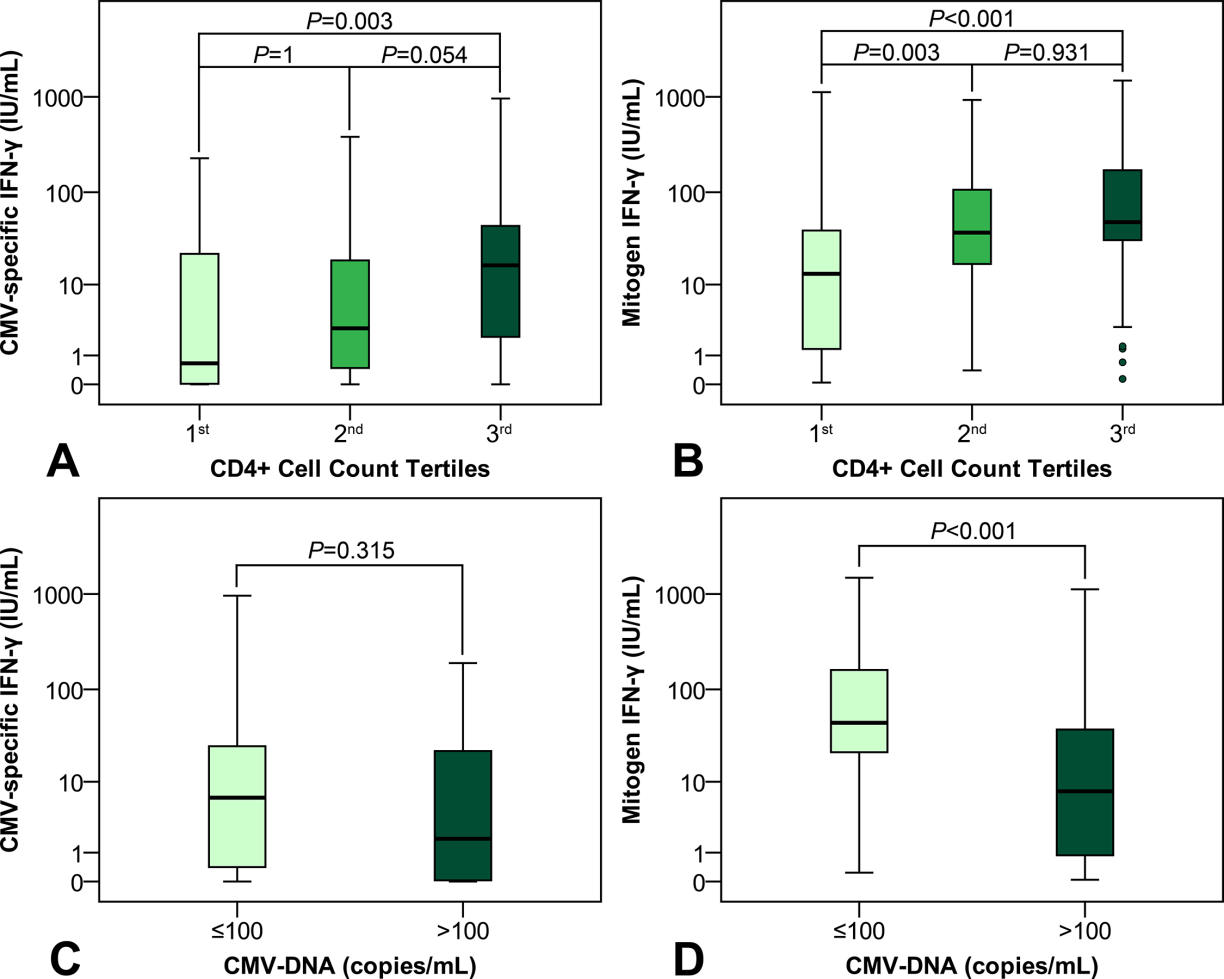
Tukey’s schematic boxplot showing the association between CMV-specific and mitogen IFN-gamma response and both CD4^+^ (A, B) and CD8^+^ (C, D) T cell count. CD4^+^ T cell count tertiles: 1^st^: < 115, 2^nd^: 115–217, and 3^rd^: > 217 cells/μL. CD8^+^ T cell count tertiles: 1^st^: < 478, 2^nd^: 478–903, and 3^rd^: > 903 cells/μL.

Individuals were grouped according to CD4^+^ T cell count tertiles (1^st^: < 115, 2^nd^: 115–217, and 3^rd^: > 217 cells/μL). CMV-specific IFN-gamma response significantly varied throughout the CD4^+^ T cell count tertiles (*P* = 0.003) (Fig 2). Post-hoc comparisons revealed, that patients in the 3^rd^ tertile (16.5 (IQR 53.9) IU/mL) had a higher CMV-specific IFN-gamma response than patients in the 1^st^ tertile (0.658 (IQR 23.78) IU/mL; *P* = 0.003). While there was a trend towards a higher CMV-specific IFN-gamma response in patients in the 3^rd^ tertile, when compared to patients in the 2^nd^ tertile (2.84 (IQR 19.07) IU/mL; *P* = 0.054), the responses of subjects in the 1^st^ and the 2^nd^ tertile were comparable (*P* = 1).

Moreover, we observed higher mitogen IFN-gamma responses with higher CD4^+^ T cell counts (*P* < 0.001) (Fig 2). Mitogen IFN-gamma responses were higher in patients in the 2^nd^ (37.3 (IQR 90.2) IU/mL; *P* = 0.003) and the 3^rd^ tertile (48.2 (IQR 149.8) IU/mL; *P* < 0.001), when compared to patients in the 1^st^ CD4^+^ T cell count tertile (13.3 (IQR 38.4) IU/mL). Patients in the 2^nd^ and 3^rd^ tertile showed similar mitogen IFN-gamma responses (*P* = 0.931).

### Impact of CD8^+^ T cell count on CMV-specific and mitogen IFN-gamma response

Patients were divided into subgroups according to CD8^+^ T cell count tertiles (1^st^: < 478, 2^nd^: 478–903, and 3^rd^: > 903 cells/μL). We observed higher CMV-specific IFN-gamma responses with higher CD8^+^ T cell counts (*P* = 0.033) (Fig 2). Patients in the 3^rd^ CD8^+^ T cell count tertile (15.11 (IQR 72.04) IU/mL) had higher CMV-specific IFN-gamma responses when compared to patients in the 1^st^ tertile (2.78 (IQR 17.6) IU/mL; *P* = 0.034), while the difference between patients in the 3rd and the 2nd (10.44 (IQR 29.3) IU/mL; *P* = 1), or the 2nd and the 1st (*P* = 0.206) CD8^+^ T cell count tertile did not attain statistical significance.

Mitogen IFN-gamma response significantly varied throughout the CD8^+^ T cell count tertiles (*P* < 0.001) (Fig 2). Mitogen IFN-gamma responses observed in the 3^rd^ CD8^+^ T cell count tertile (89.34 (IQR 178.59) IU/mL) were higher than mitogen IFN-gamma responses observed in both the 1^st^ (23.33 (IQR 51.7); *P* < 0.001) and the 2^nd^ (35.31 (IQR 89.33) IU/mL; *P* = 0.011) CD8^+^ T cell count tertile. In contrast, mitogen IFN-gamma responses were comparable between the 2^nd^ and the 1^st^ tertile (*P* = 0.211).

### Independent associations between CD4^+^ and CD8^+^ T cell counts on CMV-specific IFN-gamma response

There was a direct correlation between CD4^+^ and CD8^+^ T cell counts (ρ = 0.372; *P* < 0.001) (Table 3). Furthermore, we observed a correlation between CD4^+^ (ρ = 0.219; *P* = 0.009) and CD8^+^ T cell counts (ρ = 0.247; *P* = 0.003) and CMV-specific IFN-gamma responses. To assess, if there was an independent association between CD4^+^ T cell count and CMV-specific IFN-gamma response, we performed a partial correlation analysis controlling for CD8^+^ T cell counts. Interestingly, the correlation between CD4^+^ T cell count and CMV-specific IFN-gamma response did not attain statistical significance (ρ = 0.142; *P* = 0.097). In contrast, we observed an independent correlation between CD8^+^ T cell count and CMV-specific IFN-gamma response (ρ = 0.401; *P* < 0.001).

**Table 3:** Correlations between CD4^+^ and CD8^+^ T cell counts, as well as CMV-specific and mitogen IFN-gamma responses.

Patient characteristics	CMV-specific IFN-gamma response		Mitogen IFN-gamma response	
	ρ	*P* value	ρ	*P* value
CD4^+^ T cell count	0.219	0.009	0.363	< 0.001
CD4^+^ controlled for CD8^+^ T cell count	0.142	0.097	0.226	0.007
CD8^+^ T cell count	0.247	0.003	0.482	< 0.001
CD8^+^ controlled for CD4^+^ T cell count	0.183	0.031	0.401	< 0.001

CMV = cytomegalovirus

### Relationship between CMV-DNAemia and CMV-specific as well as mitogen IFN-gamma response

**Fig 3:**
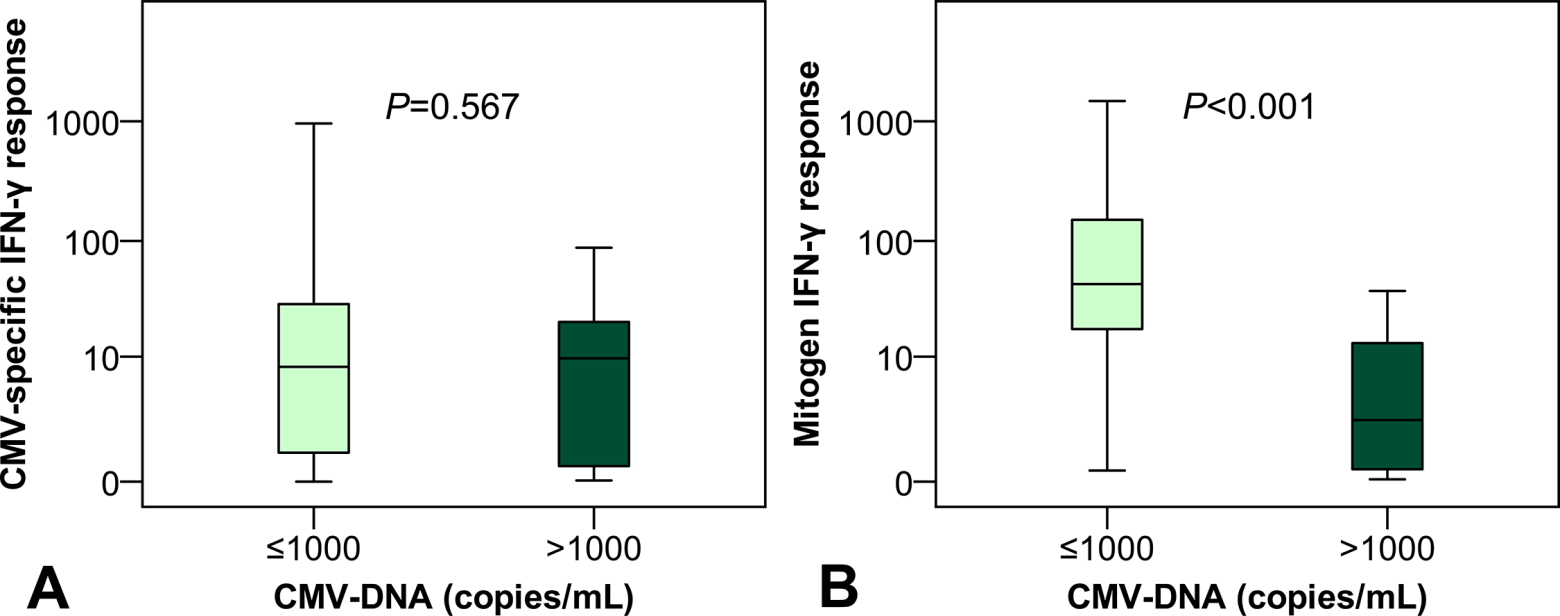
Tukey’s schematic boxplot showing the association between CMV-specific (A) and mitogen (B) IFN-gamma response and CMV-DNAemia.

While CMV-specific IFN-gamma responses were comparable between patients with (9.91 (IQR 21.58) IU/mL) and without (8.03 (IQR 28.54) IU/mL; *P* = 0.567) CMV-DNA > 1000 copies/mL, a statistically significant inverse association between mitogen IFN-gamma response and significant CMV-DNAemia was observed (*P* < 0.001) (Fig 3). Patients with CMV-DNA ≤ 1000 copies/mL (43.2 (IQR 134.6) IU/mL) had higher mitogen IFN-gamma responses when compared to patients with CMV-DNA > 1000 copies/mL (2.26 (IQR 17.95) IU/mL).

### Cases of CMV end-organ manifestation during follow-up

During the observational period, a total of 5 individuals (3.3%; 5/153) were diagnosed with CMV-related end-organ manifestation with a median delay of 210 days from inclusion. The mean CD4^+^ T cell count was 22 ± 9 cells/μL and two subjects were on ART at the time of study inclusion. Four patients had CMV retinitis and one subject was diagnosed with CMV oesophagitis. One of the five individuals was seronegative for both CMV IgG and IgM. All five subjects had detectable CMV DNAemia with 4/5 patients displaying CMV DNA levels > 1000 copies/mL. The mean CMV DNA level was 289337 copies/mL. One of these individuals was CMV-QFT reactive and CMV-QFT negative, respectively, while three individuals displayed CMV-QFT indeterminate results.

## Discussion

The majority of HIV-1–infected individuals is seropositive for CMV [14] with the seroprevalence rate among HIV-1–infected homosexual men being reported to be as high as 95–100% [15]. Reactivation of CMV replication and CMV end-organ manifestation occur in individuals with impaired T cell function. HIV-1–infected subjects with advanced immunosuppression are thus at increased risk for developing CMV disease. Effective cellular immune responses, in particular involving CD8^+^ T cells and natural killer (NK) cells, are a prerequisite for the recovery from infection with CMV [16]. Cytomegalovirus-specific IFN-gamma production is associated with protection against cytomegalovirus reactivation in HIV-1–infected patients [17]. The recently developed CMV-QFT measures the production of IFN-gamma following stimulation with previously defined CD8^+^ T cell CMV epitopes and is used to assess the presence of CMV-specific immunity.

Only a limited number of studies assessing the value of CMV-specific IFN-gamma release assays (IGRA) have been published to date. These studies were predominantly performed in solid-organ transplant recipients [11,18–22] and in subjects who underwent allogeneic stem cell transplantation [23–26]. Manuel et al. [11] showed that among donor CMV-seropositive, recipient-seronegative solid-organ transplant recipients, those individuals with a positive CMV-QFT result subsequently had a lower incidence of CMV disease than patients with negative and indeterminate results. The highest incidence of CMV disease was observed in subjects with an indeterminate CMV-QFT result. Similar results were reported in a recent study by Weseslindtner et al. [21] in 67 lung transplant recipients. In the latter study, the incidence of subsequent CMV viremia was significantly lower in patients with positive when compared to negative CMV-QFT results.

CMV replication has been identified as a significant cause of immune activation in HIV-1–infected subjects [27,28]. Among HIV-1-infected individuals, an increased T cell response to CMV antigens has been described which may be linked to intermittent viremia and antigen exposure [29]. In HIV-1–infected subjects with advanced immunosuppression, i.e. defined as CD4^+^ T cell count ≤ 50 cells/μl, CMV-specific immunity measured by IFN-γ-ELISPOT was associated with a delayed development of CMV viremia and end-organ manifestations [30]. So far, only one study has investigated the use of the CMV-QFT in the HIV-1 Infected. Singh et al. [31] found that subjects with previous CMV manifestations had reduced CMV-specific CD8^+^ T cell responses, when compared to patients without a history of CMV manifestation. Similar to this study, mitogen response as compared to CMV-specific response was more strongly associated with CD8^+^ T cell count in our study participants. However, the retrospective design and the low number of patients included in the study by Singh et al. limit their conclusions on the value of the CMV-QFT assay. Moreover, CMV-specific CD4^+^ T cell responses—which also play a role in the control of CMV infection—increase with ART and are more pronounced at the time of advanced immunosuppression [32].

To the best of our knowledge, our study is the first to longitudinally assess the performance of the CMV-QFT in a cohort of individuals with advanced HIV-1 infection. In the current study, we performed the CMV-QFT assay in HIV-1–infected individuals with advanced immunosuppression defined as a current CD4^+^ T cell count ≤ 350 cells/μl. The proportion of patients with CMV-DNAemia > 100 copies/mL was significantly higher in CMV-QFT non-reactive patients, when compared to CMV-QFT reactive individuals. However, a significant proportion of subjects who developed CMV end-organ manifestation had indeterminate results in which both mitogen and CMV antigen responses are absent. This is likely caused by the advanced HIV-1–induced immunosuppression in these patients. Thus, the dependency of CMV-QFT results upon actual CD4^+^ and CD8^+^ T cell counts should be considered and taken into account when interpreting CMV-QFT results in HIV-1–infected individuals. This is in accordance with previous studies on the tuberculosis-specific IGRA showing impaired test performance in individuals with reduced CD4^+^ T cell counts [33,34]. However, the association between CD4^+^ T cell count and CMV-specific IFN-gamma responses may be partially explained by the correlation between CD8^+^ T cell count and both variables, as correlation between CD4^+^ T cell count and CMV-specific IFN-gamma response did not attain statistical significance, after controlling for CD8^+^ T cell count. Furthermore, the association of CMV disease development with indeterminate CMV-QFT results has also been reported in solid-organ transplant recipients [11]. All subjects who developed a CMV end-organ manifestation had CD4^+^ T cell counts of less than 50 cells/μL. This cut-off has been defined based on the results of previous studies assessing the risk of CMV end-organ manifestations in HIV-1–infected individuals [30]. Therefore, the CMV-QFT might provide additional, clinically relevant information in these patients. However, the low number of patients with CD4^+^ T cell counts of less than 50 cells/μL included in our study limits the conclusions drawn from this observation. The actual clinical value of additional CMV-QFT testing remains to be established, since the ‘standard’ CD4^+^ T cell count determination might already indicate an increased risk for CMV infection in patients with low CD4^+^ T cell counts. Further studies are warranted to assess the relevance of CMV-QFT in this setting.

This study had several limitations. We analyzed not only clinically symptomatic CMV disease cases but individuals with CMV-DNAemia due to the low incidence of CMV end-organ manifestations in our study. Thus, future studies assessing predictive value of the CMV-QFT for the development of CMV end-organ manifestations should be performed in adequate at risk populations, e.g. patients with CD4^+^ T cell counts of less than 50 cells/μL. Furthermore, the manufacturer recommends multiple tests at different time points, as it may improve test performance. However, sequential CMV-QFT testing was not performed in our study. In addition, comparison with a control group consisting of HIV-1-uninfected subjects would have added interesting information.

In conclusion, the CMV-QFT might be useful to assess T-cell mediated immunity in HIV-1–infected subjects. Therefore, it might be used to tailor the use of CMV-specific therapy or prophylaxis. While CMV-QFT reactivity indicates CMV-specific immunity, indeterminate results due to negative mitogen IFN-gamma response might reflect HIV-1-induced immunodeficiency. Importantly, in the at risk population of patients with CD4^+^ T cell counts of less than 50 cells/μL, indeterminate CMV-QFT results might identify a subgroup of patients at highest risk for the development of CMV end-organ manifestations. Moreover, clinicians should consider the dependency of CMV-QFT results upon CD4^+^ and CD8^+^ T cell count when interpreting CMV-QFT results.

## Supporting Information

S1 File STARD Checklist. (DOC)
